# Big data collection in pharmaceutical manufacturing and its use for product quality predictions

**DOI:** 10.1038/s41597-022-01203-x

**Published:** 2022-03-23

**Authors:** Janja Žagar, Jurij Mihelič

**Affiliations:** 1 Lek Pharmaceuticals d.d., Ljubljana, Slovenia; 2 University of Ljubljana, Faculty of Computer and Information Sciences, Ljubljana, Slovenia

**Keywords:** Chemical engineering, Chemical physics, Design, synthesis and processing, Characterization and analytical techniques

## Abstract

Advances in data science and digitalization are transforming the world, and the pharmaceutical industry is no exception. Multiple sensor-equipped manufacturing processes and laboratory analysis are the main sources of primary data, which have been utilized for the presented dataset of 1005 actual production batches of selected medicine. This dataset includes incoming raw material quality results, compression process time series and final product quality results for the selected product. The data is highly valuable for it provides an insight into every 10 seconds of the process trajectory for 1005 actual production batches along with product quality collected over several years. It therefore offers an opportunity to develop advanced analysis models and procedures which would lead to the omission of current conventional and time consuming laboratory testing. Benefits for both the industry and patient are obvious: reducing product lead times and costs of manufacture.

## Abstract (summary)

**Table:** 

Measurement(s)	Incoming raw material quality (particle size distribution, water content, impurities level, residual solvents, pH) • In process control measurements of tablet core and film coated tablets (weight, thickness, diameter, hardness, yield of a process) • Final medicine quality on a representative sample of film coated tablets (drug release in defined time, active ingredient content, impurities level, residual solven content) • Process time series of selected tablet compression parameters (every 10 s of the compression process)
Technology Type(s)	Laboratory based analysis (particle sizer using laser diffraction, loss on drying method, HPLC method, GC method) • Automatic IPC check machine (combining balance, hardness, thickness and diameter measurements) • HPLC (High performance liquid chromatography), GC (gas chromatography) • Tablet compression machine calibrated sensors for the following main parameters: main and pre-compression force, fill depth, cylindrycal height, ejection force, number of wasted tablets.
Factor Type(s)	batch • code • strength • size • start • api_code • api_batch • smcc_batch • lactose_batch • starch_batch • api_water • api_total_impurities • api_l_impurity • api_content • api_ps01 • api_ps05 • api_ps09 • lactose_water • lactose_sieve0045 • lactose_sieve015 • lactose_sieve025 • smcc_water • smcc_td • smcc_bd • smcc_ps01 • smcc_ps05 • smcc_ps09 • starch_ph • starch_water • tbl_min_hardness • tbl_max_hardness • tbl_av_hardness • tbl_min_thickness • tbl_max_thickness • fct_min_thickness • fct_max_thickness • tbl_min_weight • tbl_max_weight • tbl_rsd_weight • fct_rsd_weight • fct_min_hardness • fct_max_hardness • fct_av_hardness • tbl_tensile • fct_tensile • tbl_yield • batch_yield • time series: tbl_speed • time series: fom • time series: main_comp • time series: tbl_fill • time series: SREL • time series: pre_comp • time series: produced • time series: waste • time series: cyl_main • time series: cyl_pre • time series: stiffness • time series: ejection
Sample Characteristic - Organism	Selected medicine
Sample Characteristic - Environment	manufacturing process
Sample Characteristic - Location	Pharmaceutical industry

## Background & Summary

The pharmaceutical industry is known for innovation, development of life-saving products, and a high level of quality^1^. On the other hand, it is also relatively reluctant to embrace changes, new technologies, and approaches that could improve ways of working in the production of medicines^2,3^. Why? Primarily due to the highly regulated field, which calls for a long and document-heavy process of change implementation^4^.

Regulatory bodies such as FDA (Food and Drug Administration, US) and EMA (European Medicines Agency) have acknowledged that a shift towards more data-oriented medicine manufacturing needs to occur^5,6^. The pharmaceutical industry is being encouraged to embrace new digital technologies and better utilize the data collected for the demonstration of the quality of their products and improvement of manufacturing efficiency^7,8^.

Medicine manufacturing processes are equipped with numerous sensors monitoring and controlling critical process and equipment parameters^9^. Every product manufactured for the market therefore has a large amount of data describing its every step, from incoming raw materials entering into the process, process parameters, intermediate product characteristics, as well as final product quality (Table 1). These production-related datasets range from simple laboratory analysis of incoming raw materials to complex time series outputs available for every second of the manufacturing process^10,11^. The data are stored in several different databases and servers and are normally used only to confirm the predefined quality of incoming raw materials, intermediate products, and final products.

**Table 1:** Overview of data sources for a pharmaceutical product.

**Incoming materials**
Excipients’ characteristics
Active pharmaceutical ingredient characteristics
**Process**
Process time series
Intermediate product characteristics
**Final product**
Laboratory analysis
Product quality

The present data collection study focuses on a high-volume pharmaceutical product (i.e., medicine) intended for lowering blood cholesterol. The pharmaceutical dosage form is film-coated tablets with an immediate release drug profile. The product was chosen due to its simple formulation and straightforward manufacturing process.

The composition of the selected medicine consists of excipients and an active pharmaceutical ingredient (API). The excipients that were considered for this case study have a potential impact on final product quality and are as follows: lactose, silicified microcrystalline cellulose, and starch. Before their use, quality control analysis is performed for each incoming material.

The manufacturing process includes direct compression, which has a significant impact on final product quality based on expert knowledge. The process related data, therefore, consists of tablet compression time series and intermediate product (i.e., tablet cores) testing data.

After the process is finalized, product quality is tested for every manufactured batch on a representative sample of film-coated tablets. The product is only released for use if all specification criteria for final product testing are met.

The data presented in this study were collected from three main databases: “Laboratory Sample Manager Database”, production database, and process time series database connected with tablet press SQL database.

Incoming materials have a significant impact on final product quality, and when combined with process data, additional insight into the product may be obtained. The dataset presented in detail below offers an opportunity for researchers to find alternative ways to determine product quality.

## Methods

Due to the sensitivity of industry data, all data descriptors (i.e., batch number, product code, excipient batches, etc.) were anonymized.

### Dataset scope

The product or product family in the scope of the research has several product sub-families, which are defined by product code. Product sub-families differ in strength and manufacturing batch size. There are four different strengths and nine different batch sizes present in the research dataset. Products of different strengths within the scope have proportional or semi-proportional formulations and only differ in the weight of the final tablet, keeping formulation ratios the same. In order to account for the differences between product sub-families, categorical data are also included in the research dataset.

The data collected for the present research range from November 2018 to April 2021. The time interval exceeding one year ensures that seasonal variation, changes in incoming raw materials, the impact of operator shift work, holidays, and other common process and equipment variability, are all taken into account. It is thus safe to assume that the presented dataset is robust and representative of the selected product.

### Data sources

The primary data sources are laboratory analysis results of incoming raw materials (excipients and API), of the intermediate product (tablet cores), and of the final product. The analyses were performed by trained laboratory technicians specialized in corresponding test. Devices used for analysis ranged from HPLC (high-performance liquid chromatography), GC (gas chromatography), moisture analyzer and particle size analyzer to automatic tablet cores analyzer.

The second primary source of data are the tablet compression process time series. Time series output, such as tablet press speed, compaction force, fill depth, etc., is generated by tablet press sensors (Table 2). Time series output is generated for every second of the process and is stored in the tablet press SQL database. From there, time series are uploaded to a server that allows for visualization or extraction of the data by domain experts. This data is semi-structured and requires cleaning and organizing before use.

**Table 2:** Sources of data in the process and analysis flow explained.

**1. Incoming raw materials**	Active pharmaceutical ingredient, excipients
1.1. Quality analysis	Water and impurities content, pH, particle size and density
**2. Process: tablet compression**	Process time series derived from tablet press sensors
2.1. Intermediate product control	Hardness, thickness, diameter, weight
**3. Final product**	Film-coated tablets
3.1. Quality analysis	Drug release, impurities content, related substances content

### Data collection methods

Before accessing and exporting securely the stored laboratory and process data, the so-called batch genealogy was performed. All laboratory and process data in the above-mentioned databases are stored using batch identifiers. In order to extract the relevant data from databases, it was necessary to determine the corresponding raw material batches that entered into each of the 1,005 final product batches included in this data descriptor study. Only after this initial information was known, did the process of data collection begin.

We exported the data by product material code (i.e., product sub-family), which groups all the batches that have been manufactured under that particular code. The export filter settings, therefore, included the time interval, product code, and laboratory analysis range.

The process time series export was more challenging compared to the laboratory data, due to the quantity of the data. The tablet compression process typically runs between 2 hours and 20 hours, depending on product sub-family (i.e., product code), which defines the batch size (i.e., the target number of tablets produced). The larger the batch size, the longer the process. We exported the data for every 10 seconds of the process and thus slightly reduced complexity, while still keeping all important process information.

The exported batch time series data provided datasets of several thousand rows (see details in the *Data Records* section). The main identifier of this type of data is a timestamp, which was unstructured and needed preprocessing due to different time formats present in the primary data.

### Preprocessing of laboratory data

The laboratory data includes the results from the incoming raw material analysis (independent variables), intermediate product quality (independent variables), and final product quality (dependent variables). We visualized every exported parameter (i.e., laboratory analysis results) to observe for any outliers.

Another aspect that needed to be evaluated was whether the data had the expected spread based on product and process knowledge. The following preprocessing steps were applied:

- Batches with no final product quality results available were excluded from the dataset.
- Four different product “sub-families” have four different target tablet core weights, which would have affected further analysis. For this reason, a normalized parameter, weight relative standard deviation (RSD), was included instead. However, for the purpose of alternative future analysis approaches, the original weight data for every batch is kept in the provided dataset.
- Every product code (sub-family) also has a different target thickness, diameter and hardness. We therefore decided to prepare a new parameter normalized with respect to tablet dimensions and shape – tensile strength (Eq. 1)^12,13^. Unlike hardness, tensile strength is a normalized parameter comparable across product sub-families. Since all product sub-families have the same cylindrical tablet shape, no alterations to Eq. 1 are needed. A new parameter was created by applying the following equation for every batch:
  1 $$Tensile\;stregth:\sigma =2\ast \frac{F}{\pi \ast t\ast d};$$
  where *F* represents tablet hardness in Newton (N), *t* is tablet thickness in millimeters (mm) and *d* represents tablet diameter in millimeters. The average hardness value and the maximum thickness and diameter for both tablet cores and film-coated tablets were considered in the calculation.
- Product quality parameters included in the dataset are final product impurities, residual solvents and drug release results.

### Preprocessing of time series data

We performed visualization on time series to evaluate the quality of data, any unusual events, and any requirements for special preprocessing or complete removal of certain batches. Among all the parameters included in the time series dataset, tablet press speed and the number of rejected tablets, counted by the press, were the most descriptive of batch dynamics. The first parameter indicates the time when batch tablet production started and whether there were any unusual process interruptions. The second parameter selected was the number of rejected tablets, because it helps to understand whether tablet press speed reaching 0 is due to the process being finished or due to another start-up or more challenging issues occurring during the process. If the number of rejected tablets does not increase, then the process is finished. Furthermore, the trajectory of rejected tablets in a batch cannot fluctuate; it can only increase with time. The following preprocessing and cleaning steps were taken:

- The process time series were quality checked by applying the above explained visualization of the two parameters for every single batch included in this dataset (1,005 batches in total).
- We have observed that some batches were stopped because of the weekends or bank holidays. Due to this, a considerable part of the time series displays the value of 0, as the tablet press was paused. When dealing with this data, this particular characteristic needs to be considered, as it could hinder future analysis. These parts have been deliberately kept as part of the time series dataset, because leaving blended powdered material to rest for prolonged periods of time could potentially impact the quality of compressed tablets.
- The tablet press SQL database includes unstructured data, and the transfer of these into a readily available database (iHistorian) resulted in a mixed, 12- and 24-hour time structure. Standardization of the time format was performed.
- Visualization enabled us to detect a drop in the rejected tablet parameter for some batches. This required a detailed data investigation for those particular batches. We discovered that this issue had occurred with batches that spread over two or more days because of the incorrect date structure in some batches. Corrections were made for all batches where the issue was detected.
- Some time series datasets had a so-called “gap”, meaning that for a period of several minutes, there were no data available, i.e., empty cells. This occurs when the tablet press is shut down for whatever reason, e.g., weekend, shift change, calibration of the tablet press, calibration of the automatic weight and hardness control system, etc. Since these gaps of missing data have no value for analysis, they were excluded.
- Once all the above steps were applied and the data has been cleaned and the time structure corrected, another visualization cycle was performed to check whether all the preprocessing steps have worked.

Only the correctly preprocessed, high quality time series data were kept and are presented in this research.

### Time series data reduction

The time series presented a vast amount of data compared to laboratory result entries for each batch. The aim was to create new parameters for each batch that would best describe the original time series data for a particular batch.

These new parameters were then combined with the laboratory data linked with the same product batch. Such data manipulation allows for further in-depth analysis, process understanding, and product quality predictions. The time series data were provided intact for this data descriptor paper as well as in a reduced format; as new attribute vectors that were tailored separately for each process parameter based on expert knowledge.

The following feature extraction steps were applied and new attributes created for every batch time series dataset:

- *Average tablet press speed:* the average speed of the tablet press machine, excluding the tablet press values of 0 due to known longer interruptions, such as weekends and holidays, which would mask the true process characteristics.
- *Number of tablet press speed changes*: tablet press speed is generally set in the beginning of the process and usually only changes or drops to 0 if issues with tablet compression arise. The best process is a constant process without interruptions or changes to the parameters. This attribute was normalized by batch size, because larger (and therefore longer) batches will naturally have more interruptions than smaller ones.
- *Tablet press speed of 0:* the total time the tablet press speed was 0. This parameter, too, was normalized by batch size for the same reason as explained above. It was necessary to consider if a batch was run or paused during the weekends and holidays. For this reason, we created an additional categorical attribute to note which batches had a “weekend run”. If the tablet press was stopped during a weekend or holiday, this time was subtracted from the total time the tablet press speed was 0.
- *Total number of rejected tablets:* this parameter is cumulative and tells us for every moment of the process how many tablets have been rejected up to a certain point. It was normalized with batch size.
- *Number of rejected tablets during start-up:* it gives an indication how much time and effort was needed to prepare tablet press parameters for a particular blend. The higher the amount of rejected tablets, the more effort was needed due to more challenging material properties or potentially an inexperienced operator. Either way, both have a potential impact on tablet quality. Normalization is not needed, because the size of the batch does not have an impact on the set-up of the tablet press.
- *Average filling device speed:* the filling device speed average excluding 0 values.
- *Filling device speed changes:* it is very uncommon to change the filling device speed during the compression process itself, so it makes more sense to consider only those changes during tablet press set-up, when the “production” parameter is 0, meaning that no good tablets have yet been made and we are therefore in the start-up phase of the process.
- *Average SREL start-up:* the average standard relative deviation of the main compression force (SREL) during compression start-up, excluding values where the tablet press is not running (i.e., the tablet press speed or the filling device speed is 0).
- *Average SREL:* the average SREL during a compression run, excluding values where the tablet press is not running (i.e., the tablet press speed or the filling device speed is 0).
- *SREL max:* the maximum value of SREL during a compression run. It is essential to eliminate any excessive values, i.e., anything above 15, because such values are unrealistic for the product based on expert product knowledge and only appear in the beginning of the tablet press run due to the main compression force transitioning from 0 to the target value. The difference is naturally large, which shows in the SREL parameter.
- Simple statistical methods were applied to other process time series parameters for the compression run as well as the start-up phase of the compression process: min, max, standard deviation, mean, median.

Based on batch sizes, normalization factors were calculated, which are included in a separate file within the enclosed dataset.

## Data Records

The data records are available at 10.6084/m9.figshare.c.5645578.v1^14^. The data is divided into the laboratory data (“Laboratory.csv”), the time series data (“Process time series”), the extracted features of time series data (“Process.csv”), and the normalization factors (“Normalization.csv”). Each of these is presented below.

### Laboratory data

This file combines genealogy information, the incoming raw materials analysis, the intermediate product analysis and the final product analysis. There are altogether 1,005 rows of data, each row presenting one final product batch (Table 3). The rows are identified by final product batch numbers; the batches can be grouped into the so-called product sub-families by product codes. Laboratory data parameters are presented and explained in Table 4.

**Table 3:** Overview of laboratory data.

Number of rows	Number of parameters	Number of categorical parameters	Number of numeric parameters	Number of independent variables	Number of dependent variables
**1,005**	**53**	**9**	**44**	**47**	**6**

**Table 4:** Detailed description of Laboratory.csv file content.

List of parameters in laboratory data file	Unit of Measure	Short description	Source of data
batch	N/A	Index column, identifies every product batch number.	**Genealogy data** *Categorical data*
code	N/A	Groups batches into so-called product sub-families defined by product code.	
strength	mg/unit	Strength of the product (i.e., mg of Active Pharmaceutical Ingredient (API) per tablet).	
size	tablets	Target number of tablets produced per batch.	
start	N/A	Starting time of production in date-time format.	
api_code, api_batch	N/A	Active pharmaceutical ingredient (API) material code and batch number.	
smcc_batch, lactose_batch, starch_batch	N/A	Silicified microcrystalline cellulose (SMCC), lactose and starch batch numbers.	
api_water	%	Content of water in API measured with loss on drying method.	**Incoming raw materials** **(lab analysis)** *Numerical data*
api_total_impurities, api_l_impurity	%	API total impurities and L impurity content, measured with High performance liquid chromatography (HPLC) method.	
api_content	%	Active ingredient content in raw material (excluding impurities, water, etc.) in %.	
api_ps01, api_ps05, api_ps09	µm	Particle diameter in microns at 10% cumulative volume (ps01), 50% (ps05), and 90% (ps09).	
lactose_water	%	Lactose water content measured with loss on drying method in %.	
lactose_sieve0045, lactose_sieve015, lactose_sieve025	%	Lactose particle size; % of weighted residual on one of the three sieves: 0.045 mm, 0.15 mm, 0.25 mm.	
smcc_water	%	Silicified microcrystalline cellulose water content in %.	
smcc_td, smcc_bd	g/ml	Silicified microcrystalline cellulose tap (td) and bulk density (bd).	
smcc_ps01, smcc_ps05, smcc_ps09	µm	Particle diameter in microns at 10% cumulative volume (ps01), 50%/ps05), and 90% (ps09).	
starch_ph	N/A	Starch pH value.	
starch_water	%	Starch water content measured with loss on drying method.	
tbl_min_thickness, tbl_max_thickness	mm	Tablet core min and max thickness measured during compression in millimeters.	**Intermediate product quality (lab analysis)** *Numerical data*
fct_min_thickness, fct_max_thickness	mm	Film coated tablets min and max thickness measured after coating in millimeters.	
tbl_min_weight, tbl_max_weight	mg	Tablet core weight minimum and maximum measured during compression.	
tbl_rsd_weight	%	Tablet core weight relative standard deviation (RSD) measured during compression.	
fct_rsd_weight	%	Film-coated tablet weight RSD measured after coating process.	
tbl_min_hardness, tbl_max_hardness, tbl_av_hardness	N	Tablet core hardness min, max and average measured during compression; in Newtons.	
fct_min_hardness, fct_max_hardness, fct_av_hardness	N	Film-coated tablets hardness min, max and average measured after coating; in Newtons.	
tbl_tensile, fct_tensile	N/A	Normalized hardness parameter calculated for tablet core and FCT: tensile strength.	
tbl_yield, batch_yield	%	Yield based on target quantity for compression process (tbl) and whole batch (batch) expressed in %.	
dissolution_av	%	Drug release from final tablet in defined time: average (calculated) % of API released in 30 minutes.	**Final product quality** **(lab analysis)** *Numerical data*
dissolution_min	%	Drug release from final tablet in defined time: minimum % of API released in 30 minutes.	
residual_solvent	%	Residual solvent content in final product measured with gas chromatography (GC) method.	
impurities_total	%	Total impurities content in final product measured with HPLC method.	
impurity_o	%	Content of impurity O in final product measured with HPLC method.	
impurity_l	%	Content of impurity L in final product measured with HPLC method.	

### Process time series data

The time series data are arranged in one file per product code (i.e., product sub-family). Each product code combines all final product batches manufactured in the selected period. The process time series data includes the most relevant tablet compression process parameters based on product history and expert knowledge collected for every 10 seconds of the process.

The following process parameters were considered: tablet press speed, filling device speed, main and pre- compression force mean value, tablet fill depth, standard relative deviation of main compression force (SREL), good and bad production, cylindrical height for main and pre- compression, bottom punch stiffness and ejection force. The effect of these parameters on tablet characteristics is summarized in Table 5, along with the shortened naming convention used in the uploaded data files as well as units for each parameter. Each time series file grouped by product code contains the same process parameters with the same naming convention.

**Table 5:** Detailed description of process time series.

Parameters in time series files	Unit of measure	Short description
timestamp	N/A	Index column; identifier of every 10 s entry.
campaign	N/A	Campaign number groups several batches (e.g., 5–15) into one manufacturing cycle; the batches belonging to the same campaign were manufactured one after the other.
batch	N/A	Batch number identifies the batch of the final product.
code	N/A	Product code number defines the product sub-family to which the batch belongs. Every time series dataset file has the same product code and contains all batches within the same product code.
tbl_speed	tablets/hour	Tablet press speed: it indicates when the process is running and when it has stopped, if there were many changes to this parameter or many stoppages, the material handling is challenging, which may indicate suboptimal product quality.
fom	rpm	Filling device speed in rotations per minute: similar to tablet press speed. If the process is running, so is the filling device. This parameter generally does not change and is only set during the start-up. If many changes (during the start-up) are observed, this again indicates potential difficulties with incoming material handling.
main_comp	kN	Main compression force – mean value: the more constant this parameter is, the more homogeneous is the incoming material blend in terms of physical properties.
tbl_fill	mm	Tablet fill depth: defines the volume of filled blended material to be compressed. If flow properties of material are poor, this parameter will vary throughout the batch and will consequently impact tablet hardness and weight.
SREL	%	Main compression force – standard relative deviation: this parameter is calculated by the tablet press itself by using main compression force mean values. It gives an indication of how uniform the tablets compacted are.
pre_comp	kN	Pre-compression force – mean value: if pre-compression force is used for tablet compaction, this parameter will be greater than 1 and will give a similar indication as main compression force. It is not readily used for the product in the scope.
produced	tablets	Good production: all acceptable tablets that have been produced at that particular timestamp.
waste	tablets	Bad production: tablets that do not pass the set tablet press parameters (i.e., max % deviation from the set main compression force – mean value). This is also a cumulative parameter and gives information about all rejected tablets at that particular time.
cyl_main	mm	Cylindrical height – main compression: cylindrical height of the tablet (main compression station) in mm. The height and hardness of the tablet are changed by changing the cylindrical height.
cyl_pre	mm	Cylindrical height – pre-compression: cylindrical height of the tablet (pre-compression station) in mm.
stiffness	N	Bottom punch stiffness in Newton: when the limit is reached, the press is stopped with suitable diagnosis. An equipment parameter.
ejection	N	Maximum tablet ejection force: if this parameter rises, the tablet ejection friction is higher, which could mean that some minor sticking of the tablet has occurred on the tablet tooling.

It should be noted that all potential variations of the process parameters described in the table below led to a process within specification limits for all batches included in this research dataset. The variation of process parameters and product characteristics within the specification limits is normal and expected.

### Extracted features of time series data

The time series data needed reduction and creation of new attributes before they could be readily used for the prediction analysis selected for the product. An example of attribute preparation is detailed in the *Methods* section, *Preprocessing of time series data*. A list of new attributes replacing the whole time series per each batch is detailed in Table 6.

**Table 6:** Description of attributes derived from time series per batch.

New time series-derived attributes	Unit of Measure	Short description
tbl_speed_mean	tablets/hour	Mean tablet speed excluding tablet speed values of 0.
tbl_speed_change	N/A	Number of changes of tablet speed, normalized with batch size.
tbl_speed_0_duration	N/A	Duration of tablet speed at 0, normalized with batch size; weekends excluded.
total_waste	tablets	Total number of rejected tablets per batch, normalized with batch size.
startup_waste	tablets	Total number of rejected tablets during the start-up of the tablet press.
weekend	N/A	Weekend batch run: categorical variable (yes/no).
fom_mean	rpm	Mean value of filling device speed, excluding time when tablet press speed was 0.
fom_change	N/A	Number of filling device speed changes (during the start-up).
SREL_startup_mean	%	Mean standard relative deviation of main compression force (SREL) value during the start-up phase of the compression process.
SREL_production_mean	%	Mean SREL value during the production phase of the compression process.
SREL_production_max	%	Max SREL value during the production phase of the compression process.
main_CompForce mean	kN	Main compression force mean value during the production phase of the process.
main_CompForce_sd	kN	Main compression force standard deviation during the production phase of the process.
main_CompForce_median	kN	Main compression force median during the production phase of the process.
pre_CompForce_mean	kN	Pre-compression force mean value during the production phase of the process.
tbl_fill_mean	mm	Tablet fill depth volume mean value during the production phase of the process.
tbl_fill_sd	mm	Tablet fill depth volume standard deviation during the production phase of the process.
cyl_height_mean	mm	Cylindrical height mean value during the production phase of the process.
stiffness_mean	N	Mean bottom punch stiffness during the production phase of the process.
stiffness_max	N	Max bottom punch stiffness during the production phase of the process.
stiffness_min	N	Min bottom punch stiffness during the production phase of the process.
ejection_mean	N	Ejection force mean value (production phase of the process).
ejection_max	N	Ejection force max value (production phase of the process).
ejection_min	N	Ejection force min value (production phase of the process).
Startup_tbl_fill_maxDifference	mm	Maximum difference between min and max tablet fill depth value (during the start-up phase of the process).
Startup_main_CompForce_mean	kN	Main compression force mean value during the start-up phase.
Startup_tbl_fill_mean	mm	Tablet fill depth mean value during the start-up phase.

Based on batch sizes, normalization factors were calculated, which are included in a separate file within the enclosed dataset.

## Technical Validation

The laboratory data included in the current study are generated by trained laboratory technicians. Each analysis is performed following approved operating procedures for a particular test. The equipment used for the analysis is qualified by the supplier and the engineering team before release for use in the company. The maintenance of these analytical instruments demands periodic services by an external certified company and regular calibration before use for analysis. Analytical instruments need to comply with strict international and internal industry standards and are subject to regular audits, which confirm the robustness and reliability of these devices. Every analytical result generated as described above is then transcribed into a dedicated database by a laboratory technician that performed the analysis. This entry needs to be verified and signed off by a second person in order for it to be uploaded into the database. The entire process follows good laboratory, manufacturing and documentation practice as defined by pharmaceutical industry standards (international and internal), resulting in data we can trust.

**Fig. 1:**
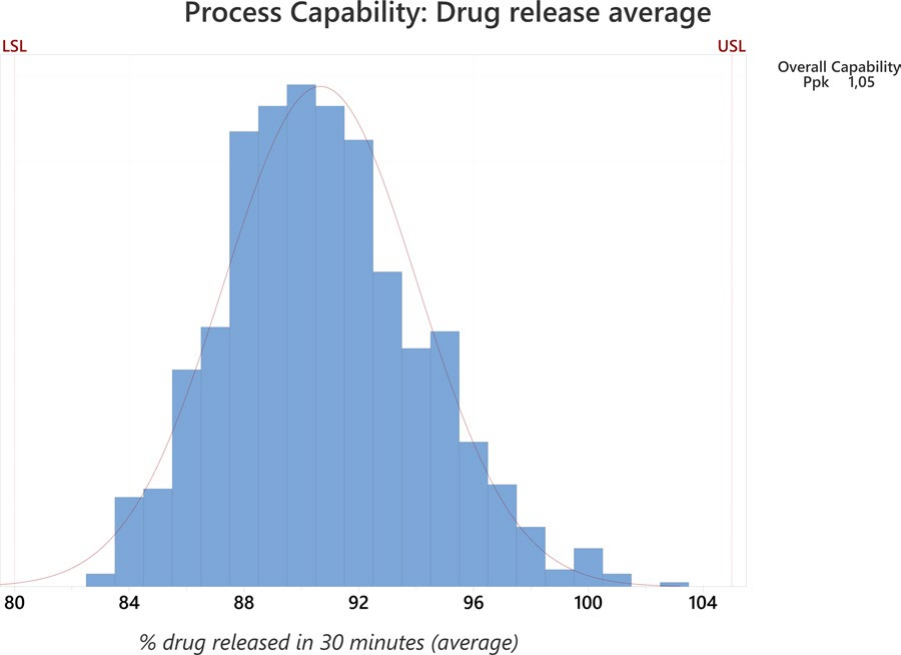
Process capability graph and Ppk calculation for drug release average, where the X-axis shows the % of drug released and the Y-axis the % of all results.

**Fig. 2:**
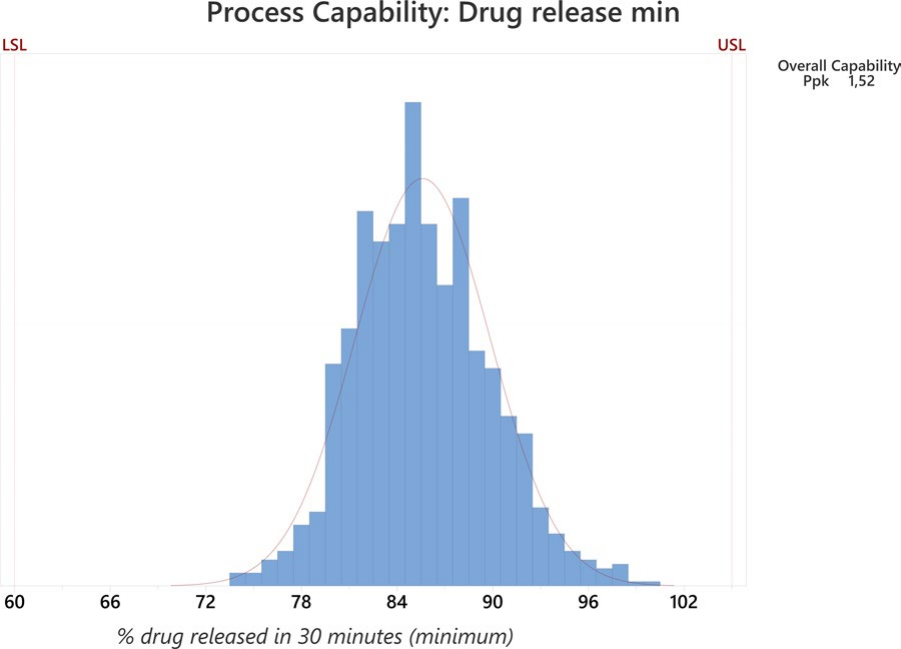
Process capability graph and Ppk calculation for drug release minimum, where the X-axis shows the % of drug released and the Y-axis the % of all results.

The laboratory data for 1,005 batches have been collected from databases and verified further to check for potential outliers and observe whether the scatter of the data is expected for the selected product. We applied statistical analysis to all collected laboratory parameters. Furthermore, the process capability index was calculated for measurements where laboratory data follows normal distribution and the parameter includes both the upper and the lower specification limit. (Note: quality parameters often only have either the upper or the lower specification limit, not both.) Overall process capability (Ppk) tells us whether the process is under control and within the limits (i.e., upper and lower specification limits). Ppk above 1 indicates a well-controlled process and is expected for the selected product (e.g., Figs. 1, 2). The laboratory parameters that are applicable for Ppk calculation are dissolution average (Fig. 1) and dissolution min (Fig. 2), which both demonstrate normal distribution. This is confirmed by a comparison of the actual process data (blue histogram bars) with a normal distribution curve (solid red curve on the graph). Ppk calculation applied to these data is as follows:

2 $$Ppk=\min [(USL-\bar{X})/3\sigma ],[(\bar{X}-LSL)/3\sigma ];$$

where USL stands for the upper specification limit, LSL the lower specification limit, $\bar{X}$ for the average value and σ for standard deviation. Statistical evaluation results (average, standard deviation, minimum and maximum values), which are gathered in Table 7 and indicate the data, follow the expected spread without extreme outliers from the average.

**Table 7:** Statistical analysis and performance qualification.

List of parameters in laboratory data file (units of measure)	Average	Standard deviation	Relative standard deviation (%)	Min	Max
API (Active pharmaceutical ingredient) water (%)	1.5	0.4	29.7	0.0	2.7
API total impurities (%)	0.2	0.1	50.1	0.1	0.5
API L impurity (%)	0.1	0.0	35.3	0.0	0.1
API content (%)	94.4	0.4	0.4	93.3	95.6
API particle size 0.1 (µm)	2.7	1.2	45.6	0.0	6.0
API particle size 0.5 (µm)	39.7	11.7	29.6	8.3	67.0
API particle size 0.9 (µm)	159.7	25.1	15.7	79.1	232.0
Lactose water (%)	0.1	0.0	14.6	0.0	0.1
Lactose sieve 0.045 mm (%)	17.5	1.1	6.2	15.0	19.0
Lactose sieve 0.15 mm (%)	50.4	1.6	3.1	44.0	53.0
Lactose sieve 0.25 mm (%)	82.3	1.2	1.5	80.0	86.0
SMCC (silicified microcrystalline cellulose) water (%)	4.5	0.1	3.1	4.3	4.7
SMCC tap density (g/ml)	0.4	0.0	2.1	0.4	0.5
SMCC bulk density (g/ml)	0.3	0.0	3.3	0.3	0.4
SMCC particle size 0.1 (µm)	32.5	1.8	5.5	30.4	37.6
SMCC particle size 0.5 (µm)	120.1	4.5	3.7	111.4	126.8
SMCC particle size 0.9 (µm)	257.8	7.8	3.0	236.8	270.2
Starch pH	4.5	0.1	3.0	4.3	4.8
Starch water (%)	2.6	0.6	24.3	1.8	3.9
Tablet core min and max thickness (mm)	A statistical analysis of these parameters is not applicable due to different target values for diameter, thickness, hardness and weight across four product sub-families. These data are included in a normalized parameter: tensile strength as explained before. The data are nonetheless provided in the dataset in case other researchers attempt to use them differently.				
FCT (film coated tablet) min and max thickness (mm)					
Tablet core weight min, max (mg)					
Tablet core RSD (%)					
FCT weight RSD (%)					
Tablet core hardness min, max, average (N)					
FCT hardness min, max, average (N)					
Tablet core tensile strength	1.3	0.3	24.1	0.8	2.4
FCT tensile strength	1.7	0.4	22.3	1.0	3.0
Tablet press yield (%)	98.3	1.1	1.1	88.0	100.8
Batch yield (%)	98.3	1.1	1.1	88.0	100.9
Drug release average (%)	90.6	3.4	3.7	82.5	102.7
Drug release min (%)	85.6	4.2	4.9	74.0	100.0
Residual solvent (%)	0.0	0.0	91.1	0.0	0.2
Total impurities (%)	0.1	0.1	71.3	0.1	0.6
Impurity O (%)	0.1	0.0	17.7	0.0	0.2
Impurity L (%)	0.1	0.0	40.9	0.1	0.2

All measurements are within the specification limits for each parameter. The greatest variation occurs for API total impurities, residual solvent and total product impurities (RSD is 50.1%, 91.1% and 71.3% respectively). This variation is acceptable due to the small absolute spread of the data. Furthermore, the upper specification limit for these three parameters is double or higher the maximum value, which further confirms the acceptability of results given.

The time series data included in the current study are generated by the tablet press machine. Tablet presses are installed and qualified by the supplier. Before they are released for use in production, an internal engineering team performs extensive qualifications and verifies all operational functionalities that need to comply with the predefined user requirements. Tablet presses are then approved for use in production and are regularly serviced, re-qualified and calibrated with the frequency defined by international pharmaceutical standards for production equipment. Software and server functionalities are separately verified on a regular basis by dedicated IT teams. Tablet presses are also subject to regular audits, which demand the highest industry standards for every production equipment. The data generated by the tablet press are thus a reliable source of information about process dynamics.

**Fig. 3:**
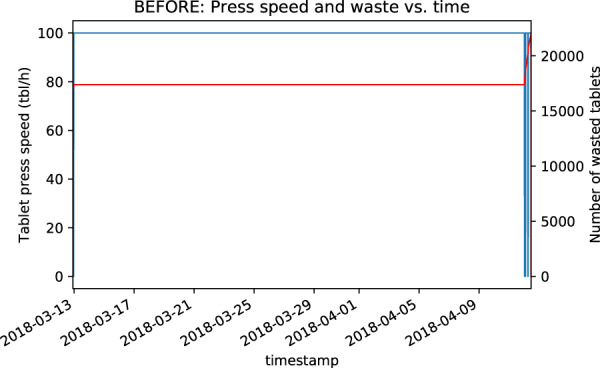
Visualization example of process time series before preprocessing.

**Fig. 4:**
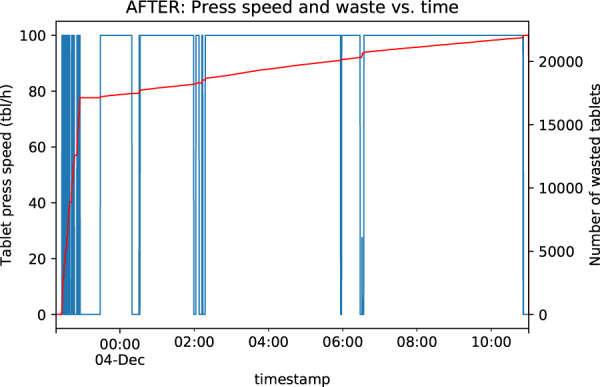
Visualization example after preprocessing (time format correction).

The time series were extracted from a server linked with the tablet press database and visualized. In total, 1,005 visual inspections were carried out as part of initial data validation. The batches where process profiles did not follow the expected trend were inspected in detail and preprocessed if needed. An example of a preprocessing requirement due to unstructured time may be seen in Figs. 3 and 4.

Besides unstructured time for some batches (e.g., Fig. 3), there was a so-called “data gap” observed. These are short intervals of several minutes, where, for some batches, no data is logged. The reason for this is loss of tablet press power (i.e., shut down). These are very rare events and were removed because they do not bring any added value to the process understanding, but could potentially impact further data analysis.

Time format correction led to time series profile, which conforms with the nature of the manufacture process in question (Fig. 4).

### Missing data

The laboratory data that are missing for some batches are active pharmaceutical ingredient (API) analysis results: total impurities, impurity L, water content and particle size. These are missing for a particular source of API material, i.e., all missing data belong to the same API code. It is recommended to either attempt further analysis with parameters that have missing data or, in case they are not relevant for the type of analysis, the parameter may be dropped altogether.

## Usage Notes

Pharmaceutical product quality analysis is mandatory before any batch of medicine is released to the market^11,15^. This process is currently predominantly laboratory based and thus very time consuming for most products across the industry. Each product batch has a wealth of data from incoming raw materials to intermediate product analysis and detailed process time series for every equipment sensor^1^. These data have been described and provided for in the present data descriptor publication for the selected pharmaceutical product. We recommend to use the data further for the prediction of final product quality (for parameters in Table 4, last 6 rows).

As described in the example analysis dataset, we have used time series datasets to extract parameters based on expert knowledge and the impact the tablet compression process has on the product in question (Table 6). If this approach does not lead to reliable enough prediction models, it would be better to use whole time series datasets and use a deep learning methodology in order to find attributes that describe the selected product quality better.

## Data Availability

Python code is available at figshare within the same collection as the dataset:^14^ 10.6084/m9.figshare.c.5645578.v1. The code is available for preprocessing, visualization and attribute extraction.
